# Health Information on Firefighter Websites: Structured Analysis

**DOI:** 10.2196/ijmr.9369

**Published:** 2018-07-16

**Authors:** Mostin A Hu, Joy C MacDermid, Shannon Killip, Margaret Lomotan, FIREWELL

**Affiliations:** ^1^ School of Rehabilitation Science McMaster University Hamilton, ON Canada; ^2^ School of Physical Therapy Western University London, ON Canada; ^3^ Roth McFarlane Hand and Upper Limb Centre St. Joseph's Hospital London, ON Canada; ^4^ Firefighter Injury/illness Remediation Enterprise: Work-participation that Enables Life & Livelihood Hamilton, ON Canada

**Keywords:** firefighters, physical health, mental health, websites

## Abstract

**Background:**

Owing to the fact that firefighters have unique health risks, access to firefighter-specific internet-based health information is a potential mechanism for achieving better health and work outcomes.

**Objective:**

The objective of our study was to identify the amount and nature of health information resources available on Canadian firefighter-specific websites and the extent to which resources are consistent across websites as a surrogate indicator of diffusion of information.

**Methods:**

A search of health resources on firefighter websites (union and employer) for all Canadian provinces, major cities and a subset of smaller cities, and the International Association of Fire Fighters (IAFF) website was conducted on Google (July 2017). Content was identified and classified based on the type of resource, health focus, and location. The quantity and nature of the resources were summarized using descriptive statistics.

**Results:**

Among all (N=313) websites reviewed, 41 websites had health information with a cumulative total of 128 resources that addressed firefighter mental (59/128, 46.1%), physical (43/128, 33.6%), and work health (26/128, 20.3%). The highest density of information was found on international and national websites (13 resources per website) and the least on local websites (1 resource per 7 websites). Three provinces (Ontario, Québec, and British Columbia) hosted 81% (65/80) of the provincial, territorial and local resources. General mental health (20/59, 34%), posttraumatic stress disorder (14/59, 24%), and suicide (14/59, 24%) were the most prevalent topics within the mental health resources, whereas half (21/43, 49%) of all physical health resources were on cancer. No resources from Northern Canada were found. Musculoskeletal health was not mentioned in any of the resources identified. There was minimal cross-linking of resources across sites (only 4 resources were duplicated across sites), and there was no clear indication of how the content was vetted or evaluated for quality.

**Conclusions:**

There was wide variation in the amount and type of information available on different firefighter websites with limited diffusion of information across jurisdictions. Quality evaluation and coordination of resources should be considered to enhance firefighters’ access to quality health information to meet their specific needs. Mental health and cancer information aligned with high rates of these health problems in firefighters, whereas the lack of information on musculoskeletal health was discordant with their high rate of work injury claims for these problems.

## Introduction

Firefighting involves complex and uncontrollable situations during fire suppression, rescues, and medical calls. These risks carry many inherent risks to mental and physical health [1]. Notable occupational hazards include exposure to carcinogens [2-4], smoke [5], high physical demands [6], and traumatic incidents [7]. The three main areas of firefighters’ health concerns are cancer [1,2], mental health issues [8-10], and musculoskeletal (MSK) injuries or cumulative trauma [11].

MSK injuries and cumulative disorders (MSKD) are not usually deadly; however, they present a predominant barrier to healthy work role participation. MSKD are the most common injuries and causes of firefighter work loss claims [1,6,12] with overexertion accounting for one-third of firefighter injury claims [13]. A study conducted by Frost and colleagues in 2013 found that MSK sprains and strains accounted for 64% of injuries reported in the Calgary Fire Department [6]. Tyakoff and colleagues [14] found that 44% of all firefighter injuries occurred on the fire scene and most were due to falls, contusions, and smoke inhalation. We found that almost half of firefighters have MSK pain localized to at least one body site [11].

A recent systematic review conducted by Jones looked at the relationship between duty-related trauma exposure and increased risk for mental health problems in first responders. The team found the accumulation of repeated traumatic exposures from calls to be a key risk factor for posttraumatic stress disorder (PTSD), depression, suicide, alcohol use, and sleep disturbances [7]. Firefighters from the United States and Canada [15] demonstrated PTSD prevalence rates of 22% and 17%, respectively, and associated the development of PTSD symptoms to traumatic incident exposures. “Dead on arrival” and “serious injury accidents” are common exposures among Canadian and US firefighters which may have cumulative effects, although the mechanisms are complex and multifactorial [16]. Firefighter-specific health information that could be reviewed online is particularly salient because firefighters have unique exposures and work within a male-dominated environment, where open discussion of mental health problems is challenging.

Another major area of health concerns for firefighters is cancer, with a meta-analysis demonstrating increased risk of multiple cancers [17], including testicular cancer, melanoma, brain cancer, esophageal cancer, and prostate cancer [18]. Owing to the fact that the increased risk of cancer is thought to be at least partially due to toxic exposures in the line of duty, awareness, prevention strategies, screening, and early management are important dimensions that can be supported by targeted health information.

Research from the Pew Internet Project conducted in 2013 discovered that 72% of internet users in the United States went online for health information [19]. Most firefighters have periods of down time and web access at their fire stations, making the web a feasible source of health information and independent learning. Further, because firefighters resist acknowledging the need for psychological help [20], the availability and anonymity of seeking health information online is especially attractive for health conditions that might have negative connotations [21].

Despite extensive research being conducted on the effects of occupational hazards on the physical and mental health of firefighters, very little attention has been paid to studying the access firefighters have to health information and how this information contributes to injury prevention and treatment. Web-based interventions for stress and anxiety management for the general population have been developed and evaluated [22,23]. However, these are unlikely to meet the needs of firefighters, given the unique and persistent exposures to critical incidents and high physical demands of urgent tasks [24,25].

Because we know that firefighters have unique information needs and are most likely to trust information coming from firefighter-specific websites, it would be helpful to understand their current access as a foundation for planning future improvements. The purpose of this study was to identify and characterize health information resources available on Canadian firefighter websites. A secondary purpose was to determine the extent to which the resources were concordant across sites as an indicator of diffusion of information across fire services.

## Methods

### Context

The population of Canada is 35,151,728, according to the 2016 Canada Census [26]. Canada is a large country geographically with a small number of large cities and large rural areas with low population. The number of professional firefighters in Canada was estimated to be 26,000 in 2016, which represents 0.72 firefighters per 1000 residents [27]. The number of volunteer firefighters was estimated to be 126,650 or 3.49 per 1000 residents [27].

### Identifying the Research Question

This structured search explored the nature and extent of available resources pertaining to the physical and mental health of firefighters by searching fire department and firefighter union websites across Canada. Relevant websites were defined as any webpages on department or union websites that contained information on the prevention, treatment, or any course of action targeting physical and mental health injuries.

### Identifying the Relevant Websites

#### Search Strategy

Google was used as the sole search engine for searches to explore resources on published fire department and firefighter union websites because this is the search engine most likely to be used by firefighters. A 3-layered search strategy (national, provincial or territorial, and local) was implemented to garner the widest scope possible while maintaining depth. In addition, because Canada is a subdivision that falls under the umbrella of the parent organization, the International Association of Fire Fighters (IAFF), the IAFF website was also included. For the national and provincial or territorial level searches, the following 3 types of unions or associations were explored: fire chiefs, professional firefighters, and volunteer firefighters. All searches were performed in July 2017.

The international and national search involved the IAFF, the Canadian Volunteer Fire Services Association, and the Canadian Association of Fire Chiefs websites. Once the association website was found, a detailed search of each page and any links on the website was conducted to identify any health-related information resources. Although many websites had information in a limited number of hierarchical levels, the IAFF website was more layered and required more extensive exploration of multiple levels to access all of the information on the website. A similar search occurred on the provincial and territorial level. Here we used Google to find the fire chiefs, professional firefighters, and volunteer firefighters association websites for the 10 provinces and 3 territories in Canada and then conducted a detailed search of each page of the website.

The initial search strategy and data extraction were developed using Ontario websites because Ontario is the largest province and has the largest proportion of the country’s population. Thus, we expected that Ontario would have the greatest number of websites. We searched for each of the 444 municipalities in Ontario, as determined by the Association of Municipalities Ontario [28]. Upon further examination of the major cities in Ontario, the search strategy was revised to look at all “single tier” municipalities (eg, Toronto, Hamilton) and “upper tier, regional” municipalities (eg, the City of Mississauga, part of the Peel region). For the rest of the municipalities in Ontario, only the major cities under counties and districts were examined (see Multimedia Appendix 1 for a detailed list of Ontario municipalities included in the search). This strategy was then extended to other provinces, although each province had a different method of organizing its municipalities. To carry a similar search methodology across provinces, the search focused on the 20 most populated cities in each province (other than Ontario), as determined by the 2016 Canadian census [26]. In provinces with regional firefighter associations encompassing smaller municipalities (New Brunswick), the regional association websites were also included in the search (see Multimedia Appendix 1 for a full list of municipalities included in the search).

#### Search Terms

For the international and national level search, the following search terms were used: IAFF, IAFF Canada, Canadian Association of Fire Chiefs, and Canadian Volunteer Firefighters Association. For the provincial and territorial level search, the following search terms were used: province or territory fire chiefs association, province or territory professional firefighters association, and province or territory volunteer firefighters association. For the local level search, once the municipalities to be included were chosen (see Multimedia Appendix 1), the following search terms were used to find websites: city or town fire chiefs association and city or town firefighters association.

### Selecting Websites

#### Inclusion and Exclusion Criteria

Official websites in English or French affiliated with a fire department or firefighter association were included. Resources were included if the firefighter website provided a link to the resource. Additional unlinked resources on the secondary website were not counted or classified. Websites under construction or inaccessible at the time of the search and social media accounts of the fire department or association were not included. This study also excluded links on firefighter websites that led to the homepages of other firefighter associations to avoid double-counting.

### Charting the Data (Health Resources)

The team developed the initial data extraction sheet and revised it based on piloting. The coding was established to classify what health problem the resource addressed, focusing on the two domains of health (physical and mental health). Because some resources were generically related to work health that could cross both physical and mental health domains, this category was added. Iterative consultation with team members was used to check consistency and subgrouping of codes. Data from the IAFF website were coded separately because it is a unique international body that has a specified mandate for firefighter health and safety. The format of the resources was classified into, for example, checklists, apps, and forums. A supplementary table (see Multimedia Appendix 2) for tools was created to collect additional information on the tools’ formats, health foci, and intended purposes.

### Collating, Summarizing, and Reporting Results

Given the large numbers of resources and codes, the data were further synthesized to characterize the major types of information available. A descriptive numerical summary (frequency distributions or percentages) was used to describe the overall number of websites included, type of websites found, types of resources, topics or focus of the resource, and location of the website publication. Summary tables were used to describe the health information resources based on type, format, and content area. The differences in the total numbers or percentages are due to rounding.

## Results

### General Findings

Overall, we found large variability in the type of health information available and the density of the information on different websites. None of the websites mentioned a process for quality appraisal or relevance vetting by firefighters to determine which type of information was posted.

### Distribution of Health Resources

**Figure 1:**
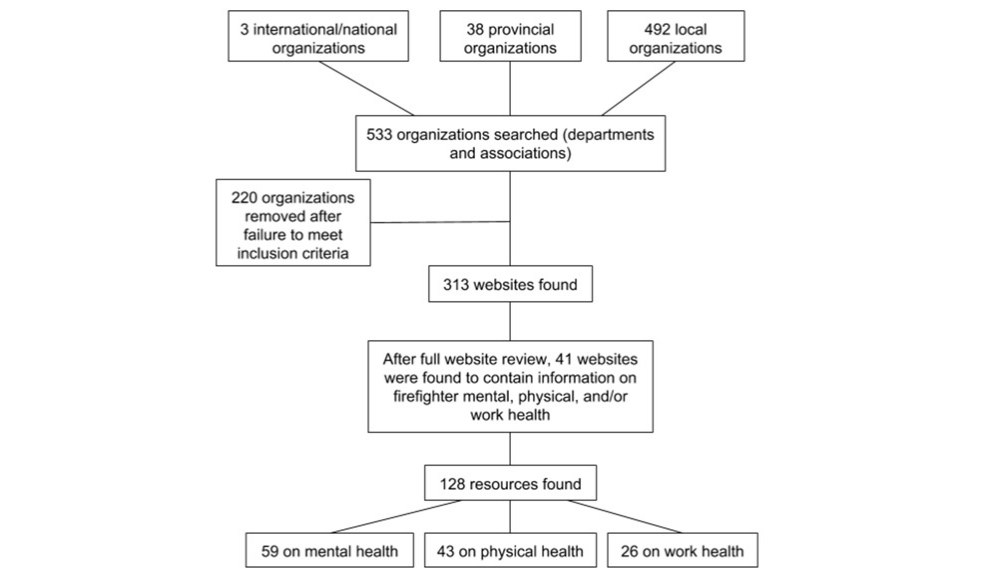
Process of website resource selection.

It was found that despite the relatively large number of associations and departments having websites (N=313), only 41 of the 313 websites (13.1%) contained health information resources relating to firefighters’ mental, physical, and work health. From these 41 websites, 128 resources were found with almost half (59/128, 46.1%) focused on mental health, a third (43/128, 33.6%) focused on physical health, and the rest (26/128, 20.3%) focused on work health, as seen in Figure 1.

**Figure 2:**
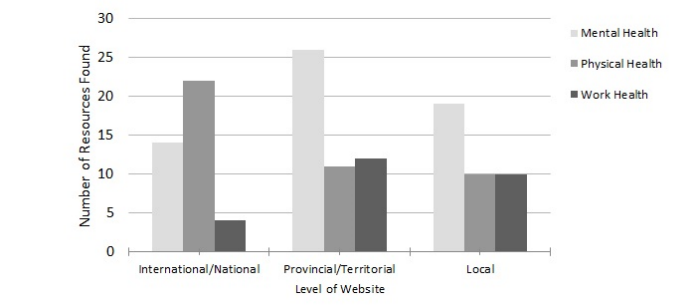
Distribution of health resources on different levels of firefighter websites.

The international and national, provincial, and local firefighter websites each contained approximately one-third of the resources found. The IAFF, national fire chief, and firefighter websites accounted for more than half (22/43, 51%) of all the resources found on physical health. The provincial and territorial and local firefighter websites focused on mental health; approximately half of all resources found on these websites addressed topics such as PTSD, critical incident stress, and suicide, as seen in Figure 2 and Table 1.

**Figure 3:**
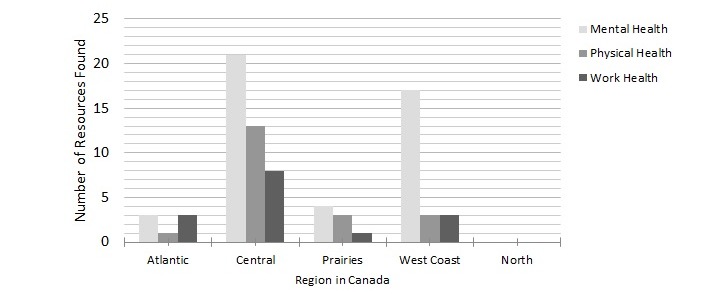
Distribution of health resources in different regions of Canada.

The resources identified in this study were predominantly found on firefighter websites from Central Canada (Québec and Ontario) and the West Coast (British Columbia). Of the total number (n=80) of resources found on the provincial and territorial and municipal level, 65 were found in these 3 provinces; of these, 42 resources were from Ontario and Québec (Central Canada) and 23 from British Columbia (West Coast). In all the regions in Canada, mental health was the most common focus on firefighter health resources. No resources of any kind were found from the firefighter websites from the Canadian Territories in the North (Yukon, Northwest Territories, Nunavut), although fire department and association websites were identified, as seen in Figure 3.

### Format and Topic of Focus

Resources on mental health (n=59) varied in format, most commonly existing as information sheets (13/59, 22%), information packets with multiple pages (9/59, 15%), and manuals (9/59, 15%; defined as larger and more structured publications with a table of contents). Articles, videos, and links to website home pages were less common, each contributing 10% (6/59) to the total number of resources identified. Many (20/59, 34%) of the mental health resources were general in topic, and there was a relatively even split between more specific topics addressed, as seen in Table 1. Resources on physical and work health were equally diverse in format, as seen in Tables 2 and 3.

A common format for physical health resources (n=43) was links to webpages (15/43, 35%), whereas a large portion of work health resources (n=26) were forms (10/26, 38%), often relating to the administrative processes involved in work injury and return-to-work (eg, workplace safety course registration; work injury or exposure claims; health insurance claims; physician statements; and return-to-work or work accommodation to track injured worker’s progress). Predominant topics addressed in physical health resources (n=43) were cancer (21/43, 49%) and health hazards (10/43, 23%). Work health resources (n=26) were most commonly on safety (12/26, 46%) and protocols (7/26, 26%).

### Density and Variation of Resources

The density of resources was highest on international and national level websites with 40 health information resources found on 3 websites (approximately 13.3 resources per website). Provincial websites averaged around 2 resources and websites with 49 resources found on 24 websites.

**Table 1:** Format and topic of focus for mental health resources (n=59).

Mental health resources		n (%)
**Format**		
	Sheet	13 (22)
	Manual	9 (15)
	Info packet	9 (15)
	Other	9 (15)
	Article	6 (10)
	Homepage	6 (10)
	Video	6 (10)
	Poster	1 (3)
**Topic of Focus**		
	General	20 (34)
	Posttraumatic stress disorder	14 (24)
	Suicide	14 (24)
	Critical incident stress	11 (18)

**Table 2:** Format and topic of focus for physical health resources (n=43).

Physical health resources		n (%)
**Format**		
	Homepage	15 (35)
	Sheet	11 (26)
	Report	7 (16)
	Video	8 (19)
	Manual	2 (5)
	Info packet	1 (2)
	Other	3 (7)
**Topic of Focus**		
	Cancer	21 (49)
	Health hazards	10 (23)
	Fitness	6 (14)
	Other	3 (7)
	Burns	2 (5)
	Reproductive	1 (2)

**Table 3:** Format and topic of focus for work health resources (n=26).

Work health resources		n (%)
**Format**		
	Form	10 (38)
	Info packet	5 (20)
	Sheet	4 (15)
	Homepage	2 (8)
	Poster	2 (8)
	Video manual	1 (4)
	Article	1 (4)
**Topic of Focus**		
	Safety	12 (46)
	Protocol	7 (26)
	Accommodation	3 (12)
	General	2 (8)
	Prevention	1 (4)
	Disability	1 (4)

Conversely, across 282 local websites, only an additional 39 health information resources were located (1 resource per 7 websites), indicating a much smaller density of information on these websites. Most of the 128 resources found were unique. Only 4 duplicate resources were found, which were mainly web links to other organizations providing relevant information (eg, asbestos.com and mesotheliomagroup.com).

### Tools

In addition to cataloging information resources, we classified resources as tools if they were structured to implement or execute specific actions or serve as a platform for engagement (eg, checklists, surveys, and mobile applications). Only a small number of tools specific to firefighters were identified. The tools (n=12) found on local websites mostly focused on physical health (10/12, 83%) with the most common format being a members’ forum (7/12, 60% of all local tools). Overall, the most common types of tools were generic or focused on work health.

**Figure 4:**
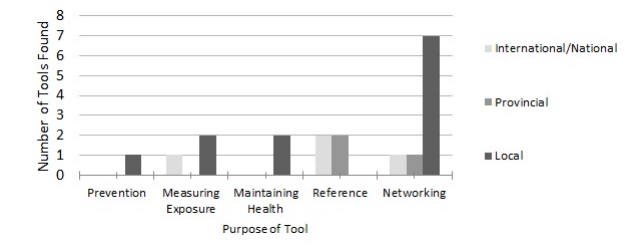
Distribution of tools on different levels of firefighter websites.

Work tools included operational resources that firefighters needed to implement workplace health and safety. The tool resources were provided in a variety of formats from forums to checklists and information packets. The majority (4/7, 57%) of national and provincial tools (n=7) were formatted as references, as seen in Figure 4.

### Trends

The IAFF website was a major source of health information specific to firefighters. In addition to health information, it provided information about in-person training courses and programs on firefighter health and safety. Provincial and local websites frequently linked to IAFF health and safety resources, as seen in Multimedia Appendices 3 and 4 in tables on IAFF mental and physical health resources.

The British Columbia (BC) Professional Fire Fighters’ website (Multimedia Appendix 5) was especially rich in mental health resources because it had a dedicated page to mental health resources that covered a wide focus, including critical incident stress management, suicide awareness, and maintaining mental health. Apart from BC’s mental health resources, Ontario had the most resources on the provincial and local levels. It had 4 provincial websites (fire chief, professional, volunteer, and women). Overall, 17 out of 102 local websites contained information and 42 out of 128 resources found were from Ontario.

In general, health information resources were most often on union, rather than employer, websites. This was particularly apparent at the local level, where almost all resources found were on firefighter association websites and very few on the municipal fire department websites.

## Discussion

### Principal Findings

This study suggests that the process by which health information is provided to firefighters through their websites is highly variable and informal in terms of a process for vetting or sharing of information. This high variability might indicate that champions for a cause, expertise in different areas, experiences within the local environment, and other factors may influence what health resources are posted on websites. The one common link for health information is IAFF, which contains the most firefighter-specific health information and resources. However, the uniqueness of information with limited crossovers between different websites indicates that there is little diffusion of information across contexts.

The density of information and provincial differences indicates that resources may be a substantial issue in determining what is shared because smaller organizations and poorer provinces had the fewest health resources. Owing to the fact that the duplication of health resource development would be a poor utilization of public dollars, greater sharing of health resources across websites or a single repository of information might facilitate effective dissemination of firefighter health information resources. Because the majority of firefighters in Canada are volunteer firefighters, with few resources and little time for face-to-face knowledge sharing, sharing of internet resources would also enhance equity in access to information. Web-based information can be shared easily across contexts, including those without the resources to create their own websites.

Given the high rate of injuries and illnesses that firefighters incur, optimizing their access and use of health information are important. Firefighter-specific websites exist for many functions beyond sharing health information. This is reflected by the fact that only 13.1% (41/313) contained any health information. Many websites, particularly city department websites, were intended for public education, providing information on the fire department, fire prevention, and outreach programs in the community. Because these are all important aspects of the fire service mandate, it is important to recognize the important competing demands that exist in terms of website development and maintenance. The development, vetting, interpretation, and application of firefighter-specific health information requires substantial resources. Labor union and association websites were more likely to provide resources that are focused directly on firefighters, which is consistent with their mandate in comparison to the fire service that has a primary mandate for public safety.

The types of resources located reflect program development and priorities in different localities. The variation in content across different cities or provinces partially reflects the difference in program development in those jurisdictions. If those programs diffused into other jurisdictions, we would have expected to find commonly shared resources on different websites. The lack of redundant information across websites indicates limited diffusion and the need for greater dissemination of information.

The fact that almost half (59/128, 46.1%) of all resources we found had a mental health focus was consistent with firefighters’ need for support to deal with higher rates of exposures to traumatic events [25]. Their day-to-day job involves situations that the general public may only see once in a lifetime [25]. Owing to this, firefighters have often noted the need for occupation-specific resources adapted for their line of work. In particular, firefighters acknowledge the importance of camaraderie and peer support in dealing with job stressors [29]. The prominence of this content reflects the recent increased awareness of these problems related to public safety personnel [9,10]. Early program development, with respect to mental health in BC (mental health resiliency programs), is reflected in the higher density of information pertaining to that topic. Although not all the elements of the mental health resiliency program may be accessible on firefighter websites, greater focus on a topic within a jurisdiction can cause firefighters to access or share more health information on this topic.

Web-based discussion forums or resources may be particularly valuable in the fire service because a community of practice or virtual support group could be established, where people share common experiences or access contextually relevant information. The advantage of using the internet for sharing mental health experiences is that people can choose to remain anonymous. A study on university students with mental health issues found that about one-third of them used the internet to deal with their mental health problems. The single most common reason cited for valuing Web-based mental health resources was that it provided anonymity [30].

Similarly, our study found that physical health resources often addressed the cancer risks due to exposure to carcinogens. Some of the few linked websites that appeared recurrently were the home pages of mesothelioma information and cancer support organizations. Mesothelioma is a rare, aggressive form of cancer that develops in the lining of the lungs, abdomen, or heart; it has no known cure and has a very poor prognosis. Firefighters are particularly at risk because exposure to asbestos is the major risk factor. A study by Daniels conducted in 2013 found a two-fold increase in malignant mesothelioma mortality and incidence in firefighters compared with that found in the general US population [31]. Given the exposure to multiple carcinogens during fire suppression and clean-up [3-5], it was unsurprising to find such a large number of cancer resources. Further, this is directly aligned with substantial literature demonstrating that firefighters are at an increased risk of different cancers. A large registry study on males with cancer found that being a firefighter was associated with higher rates of testicular cancer, melanoma, brain cancer, esophageal cancer, and prostate cancer [32]. Health information resources that encourage awareness, screening, and protection strategies could be important in mitigating these risks, for example, decontamination procedures have been widely implemented [33].

The lack of resources on MSK health stands in stark contrast to the fact that MSK injuries are among the most common injuries incurred by firefighters [6,14]. Furthermore, the lack of any resources on injury prevention on websites contrasts with the fact that substantial efforts have focused on the creation of generic MSK injury programs [34,35]. Although studies have demonstrated high rates of MSK injuries during fire suppression and training, the websites did not identify any resources specifically designed to reduce these types of injuries. It is possible that some of these resources are integrated into training programs that are delivered face-to-face or in protected sections. However, there is no reason to think that there is preferential “hiding” of MSK health resources behind firewalls in comparison to other health topics. Therefore, the lack of resources about MSK health of firefighters indicates either a research gap in that area or a lack of perceived importance within the fire service. Because MSK problems are often the most common work injury claims within the fire service [1,13,14,36], the lack of health resources in this regard indicates an important gap. The need for health information to prevent or manage MSK problems specific to firefighters is emphasized by a number of factors, which suggest that this will continue to be a substantial problem. The fire service workforce is aging, consistent with our aging workforce. Aging is associated with more MSK problems. Further studies indicate that older firefighters tend to have more MSK problems [11]. Because firefighters often start their careers with good physical health and improve their health during their early training, it can be a challenge, given the variable nature of their physical activity to maintain that high level of fitness [37]. More Web-based resources for active-duty firefighters directed toward maintaining fitness may assist with mitigating this issue. In firefighters, fitness is particularly important for preventing injuries, given the high physical demands of the occupation and the unpredictable tasks performed during fire suppression [38].

Some aspects of health that were notably absent included sleep and nutrition which are potentially important to both physical and mental health. This is a notable absence because shift work potentially impacts both of these health behaviors.

This study provides an indirect insight into the knowledge translation processes that are currently taking place with respect to health information for firefighters. Effective dissemination would be reflected in concordance or as duplicate information across websites. However, we found only four duplicates across the websites from which we extracted information. This lack of overlap is a proxy for limited dissemination. Although firefighters might be motivated to go to the IAFF website for information, recognizing it as an umbrella organization, it is unlikely that they would go to multiple websites for information. A generalized and central firefighter-specific website that features structured information sharing processes might improve dissemination of information.

Another observation was the lack of transparent processes for how information was included on websites. Both the scientific quality and the relevance of the information should be important considerations. Although these might have been considered, it was unclear to what extent this was true. Therefore, the quality of the information that is being provided to firefighters is uncertain. Of course, some resources did contain references. However, this was variable. Firefighter-specific health information is dependent on firefighter-specific health research. Generally, research that focuses on single occupations presents challenges for researchers and may not have the capacity that disease-specific health research does. This suggest that health researchers who focus on firefighter-specific topics have a heightened obligation to ensure that they engage in knowledge translation to optimize the dissemination and implementation of their work.

Regionally, Ontario was the province with the most resources available on both the provincial and local levels. This is likely because Ontario has the greatest number of firefighters in Canada with over 11,000 professional firefighters as members of the Ontario Professional Fire Fighters Association [39]. Another major contributor to the resources identified in this study was BC. This reflects substantial investments in the creation of mental health resources, despite the fact there are significantly fewer firefighters (4000) in the BC Professional Fire Fighters Association [40]. Larger organizations could be expected to have greater capacities for developing health information resources and investing in regular website updates to share those resources. Disparities in health can arise from unequal access to health information. The majority of firefighters in Canada are volunteers, and volunteer associations usually have limited resources. Because more rural areas of the country have more volunteer firefighters and small taxation bases, the likelihood of differential information access is high. Dissemination of well-developed health information in more associations has a potential to improve the health of the entire firefighter work force. Many firefighter association websites contained login-protected resources. The locked away resources potentially create a divide between volunteer and professional firefighters because many websites were members-only for firefighters who were part of the union or association. Most of the association websites identified in this study were for professional firefighters only, which means that volunteer firefighters were less likely to be able to access information on their mental, physical, and work health.

### Strengths and Limitations

This paper provided a snapshot of the Web-based health information resources from a large number of websites that may be accessed by firefighters across multiple contexts and provinces in Canada. We used a structured process to extract available resources and classify them. Although this information provides insights into the health resources available, focus, knowledge translation outcomes and gaps in the field, the nature of the work also has substantial limitations that must be considered when interpreting the results.

First, we searched only Web-based information; we recognize that this represents a fraction of the health information available to firefighters. Firefighters engage in substantial training, conferences, and other learning opportunities that likely provide additional information. Second, some of the websites had password-protected areas which we were unable to access and these areas may have contained additional resources. Third, we did not evaluate the quality of the resources.

A further limitation is that we analyzed websites and content separately, not accounting for the interdependence between the two. Further, we could not ascertain from reading websites what processes for development and vetting of resources might actually be present across different organizations. We also do not know whether firefighters are not disseminating the information or just not posting the available information on their websites. There is a possibility that firefighters shared information on social media accounts which were not included in this study.

In Ontario, only the single districts, regions, and major cities were searched, and in other provinces, the 20 most populous cities’ websites were searched; therefore, there is a chance that some resources from smaller cities or towns were missed. Although possible, this is unlikely because very little health information was found on websites for the smaller cities (eg, beyond the 10th most populated city in each province). In many of the smaller provinces, such as Prince Edward Island, New Brunswick, and Nova Scotia, there were fewer than 20 cities that existed within the entire province. Thus, all cities were included. This provided a comprehensive overview of the resources available to firefighters on fire department and association websites. Another limitation is the possibility of misclassification because a majority of the coding was conducted by a single person. To limit misclassification, the ongoing coding was examined regularly with another investigator and random rechecks of the classification were performed. Nevertheless, in classifying information, it is inevitable that the complexity of the information is lost and classifications are oversimplification of the information that was provided. An additional limitation arises from the fact that we only searched firefighter-specific websites and important information specific to firefighters could have been contained on other websites. We did this because we heard from firefighters that they tended to go to their local websites or provincial or IAFF for information. However, there are examples where substantial contributions to public safety health that would be relevant to firefighters would be contained elsewhere, for example, the Canadian Institute for Public Safety Research and Treatment recently published mental health resources (Web-based self-screening tools and research papers) for first responders [41]. The Vancouver firefighters have a critical incident stress management website [42] that is not linked to their fire department or association websites and contains information on a mental health resiliency program that was developed by Vancouver Fire and Rescue Services in collaboration with the Canadian Mental Health Association-Vancouver Fraser [43].

### Implications

The key recommendations arising from this study are the need for better coordination and sharing of health information resources specific to firefighters. Greater linkage between firefighter-specific health research and health information resources for firefighters is needed. We suggest that formal processes for evaluating what health information is shared with firefighters, widespread dissemination of lay summaries of firefighter-specific research, collaboration between researchers and firefighters, and sharing of information on formal evaluation of existing health programs would all be important contributions to effective knowledge translation. An emerging awareness of the important challenges facing firefighters with respect to firefighter health and programs was evident for mental health, whereas substantial gaps in MSK health resources were also evident. Given that some associations had few information resources, the need for greater sharing to decrease health inequities across regions is clear. Knowledge repositories and inventories that document firefighter-specific health information and those that are broadly shared might leverage existing knowledge for greater health impacts. Future research should focus on developing and evaluating the effectiveness of firefighter-specific health information resources and tools and knowledge translation plans should include widespread dissemination through firefighter websites.

## Appendix

Multimedia Appendix 1 List of Ontario municipalities included in search and list of municipalities in Canada included in search (excluding Ontario).

Multimedia Appendix 2 Tools table.

Multimedia Appendix 3 The International Association of Fire Fighters (IAFF) mental health resources table.

Multimedia Appendix 4 The International Association of Fire Fighters (IAFF) physical health resources table.

Multimedia Appendix 5 British Columbia mental health resources table.

## Abbreviations

BC: British Columbia
IAFF: International Association of Fire Fighters
MSK: musculoskeletal
MSKD: musculoskeletal disorders
PTSD: posttraumatic stress disorder
