# Supplementary material for: Health Information on Firefighter Websites: Structured Analysis
Source: Interact J Med Res. 2018 Jul 16;7(2):e12. doi: 10.2196/ijmr.9369 (PMC6066636; doi:10.2196/ijmr.9369)
Supplement: Multimedia Appendix 1 [file ijmr_v7i2e12_app1.pdf]

Appendix 1-A: List of Ontario Municipalities Included in Search

Single Tier Districts	Upper Tier Regional Districts		Major Cities in Counties and Districts
	Region	Municipality	
Brant	Durham	Oshawa	St. Thomas
Chatham-Kent		Pickering	Windsor
Greater Sudbury		Clarington	Kingston
Haldimand		Ajax	Own Sound
Hamilton		Whitby	Belleville
Kawartha Lakes		Brock	Sarnia
Norfolk		Scugog	Brockville
Ottawa		Uxbridge	London
Prince Edward	Halton	Burlington	Woodstock
Toronto		Halton Hills	Stratford
		Milton	Peterborough
		Oakville	Barrie
	Niagara	Niagara Falls	Guelph
		Port Colborne	Elliot Lake
		St. Catharines	Sault Ste. Marie
		Thorold	Timmins
		Welland	Kenora
		Fort Erie	North Bay
		Grimsby	Thunder Bay
		Lincoln	
		Niagara on the Lake	
		Pelham	
		Wainfleet	
		West Lincoln	
	Peel	Brampton	
		Mississauga	
		Caledon	
	Waterloo	Cambridge	
		Kitchener	
		Waterloo	
		North Dumfries	
		Wellesley	
		Wilmot	
		Woolwich	

Appendix 1-B: List of Municipalities in Canada Included in Search (excluding Ontario)

Province/Territory	City	Province/Territory	City
British Columbia	Vancouver	Newfoundland and Labrador	St. Johns
	Victoria		Conception Bay South
	Kelowna		Mount Pearl
	Nanaimo		Paradise
	Abbotsford		Corner Brook
	Kamloops		Grand Falls - Windsor
	Chilliwack		Gander
	White Rock		Portugal Cove - St. Phillip's
	Prince George		Happy Valley - Goose Bay
	Vernon		Torbay
	Campbell River		Labrador City
	Penticton		Stephenville
	Courtenay		Clarenville
	Duncan		Bay Roberts
	Parksville		Marystown
	Port Alberni		Deer Lake
	Cranbrook	Nova Scotia	Halifax
	Fort St. John		Cape Breton
	Terrace		Truro
	Squamish		Amherst
Alberta	Calgary		New Glasgow
	Edmonton		Bridgewater
	Red Deer		Yarmouth
	Strathcona County		Kentville
	Lethbridge		Antigonish
	Wood Buffalo		Stellarton
	St. Albert		Wolfville
	Medicine Hat	Prince Edward Island	Charlottetown
	Grande Prairie		Summerside
	Airdrie		Stratford
	Spruce Grove		Cornwall
	Leduc		Montague
	Okotoks		Kensington
	Cochrane		Alberton
	Fort Saskatchewan		Souris
	Chestermere		Georgetown
	Lloydminster	New Brunswick	Moncton
	Camrose		Saint John
	Beaumont		Fredericton

	Stony Plain		Dieppe
Saskatchewan	Saskatoon		Riverview
	Regina		Quispasmis
	Prince Albert		Miramichi
	Moose Jaw		Edmundston
	Swift Current		Bathurst
	Yorkton		Rothesay
	North Battleford		Oromoncto
	Estevan		Campbellton
	Warman	New Brunswick Regional Associations (http://www.nbafc.ca/xreg_assoc_e_db.asp)	Aboriginal Chiefs
	Weyburn		Acadian Peninsula
	Martensville		Capital District
	Melfort		Central Valley
	Humboldt		Chaleur
	Meadow Lake		Fundy
Manitoba	Winnipeg		Miramichi Valley
	Brandon		Northwest District
	Steinbach		Royal District
	Thompson	Northwest Territories	Yellowknife
	Portage la Prairie		Hay River
	Winkler		Inuvik
	Selkirk		Fort Smith
	Morden		Behchoko
	Dauphin		Fort Simpson
	The Pas		Tuktoyaktuk
	Flin Flon		Norman Wells
Québec	Montréal		Fort McPherson
	Québec		Fort Providence
	Laval		Aklavik
	Gatineau		Fort Good Hope
	Longueuil		Fort Liard
	Sherbrook		Whati
	Saguenay	Yukon	Whitehorse
	Lévis		Dawson City
	Trois-Rivières		Watson Lake
	Terrebonne		Haines Junction
	Saint-Jean-sur-Richelieu	Nunavut	Iqaluit
	Brossard		Rankin Inlet
	Repentigny		Arviat
	Drummondville		Baker Lake
	Saint-Jérôme		Cambridge Bay
	Granby		Igloolik

	Blainville		Pond Inlet
	Saint-Hyacinthe		Kugluktuk
	Mirabel		Pangnirtung
	Shawinigan		Cape Dorset
			Gjoa Haven
			Naujaat
