# Supplementary material for: Health Information on Firefighter Websites: Structured Analysis
Source: Interact J Med Res. 2018 Jul 16;7(2):e12. doi: 10.2196/ijmr.9369 (PMC6066636; doi:10.2196/ijmr.9369)
Supplement: Multimedia Appendix 2 [file ijmr_v7i2e12_app2.pdf]

Appendix 2-A: Tools Table

Developer	Tool Name	Intended User	Intended Use	Format	Mental	Physical	General	Purpose	Description
IAFF	Cancer registry	FF	R	1	0	1	0	2	part of the Wellness and Fitness initiative (WFI) by IAFF; cancer registry to report diagnosis and other info
IAFF	IAFF Frontlines App	FF	FF	3	0	0	0	6	access to event resources
Canadian Association of Fire Chiefs and Association of American Railroads	AskRail App	FR	FF, FR	3	0	0	1	5	real time information on the contents of rail cars
IAFF, linked by Alberta Fire Chiefs Association	Terrorism Response Checklist	FF, FC	FF, FC	2	1,2	4	1	5	how fire departments should prepare, handle, and recover from terrorism
Centre for Suicide Prevention, linked by BC Professional FF	First Responders Trauma Intervention and Suicide Prevention	FF, FR	FF, FR	4	1,2,3,4,6	0	1	5	info on symptoms, diagnosis, and treatment, with goal to improve mental health and prevent suicide
Newfoundland and Labrador Association of Fire Services	Member Forum	FF	FF	5	7	5	4	6	members' forum
Ontario Association of Fire Chiefs	Job Performance Checklist for Firefighters	Ff	FF	2	0	0	1	5	to be used by FF and department to ensure FF are performing the required

									skills
Ontario Association of Fire Chiefs	Job Performance Checklist for Company Officers	FF	FF (company officers)	2	0	0	1	5	details skills and duties of company officers
Hamilton Professional FF Association	Xposure	FF	WSIB, R	3	0	0	1	2	track hazards they are exposed to on calls to track chemicals/potentially carcinogenic materials
Toronto Professional FF Association	Member Forum	FF	FF	5	7	5	4	6	members' forum
Greater Sudbury Professional FF Association	Member Forum	FF	FF	5	7	5	4	6	members' forum
Norfolk Professional FF Association	Member Forum	FF	FF	5	7	5	4	6	members' forum
Ottawa Professional FF Association	PAR-Q and You	FF	FF, FC, P	1	0	4	1	4	physicians' approval before increasing physical activity
Ottawa Professional FF Association	Firefighters Medical Checklist	P	FF, FC, P	2	0	1,2,3	1,2,3	1	checklist for physicians on suggested annual tests; tracking of occupational exposures
Oshawa Professional FF Association	Member Forum	FF	FF	5	7	5	4	6	members' forum
Clarington Professional FF Association	Member Forum	FF	FF	5	7	5	4	6	members' forum
Whitby Professional FF Association	Member Forum	FF	FF	5	7	5	4	6	members' forum
Waterloo	Member	FF	FF	5	7	5	4	6	members'

Professional FF Association	Forum								forum
United Firefighters of Winnipeg	CISM Peer Support Team Contact List	FF	FF	4	2	0	1,3	4	critical incident stress management (CISM) peer support contact list
Sault Ste. Marie Professional Firefighters Association	Exposure Report	FF	P, R	1	0	5	1	2	exposure form for future reference

Legend for Appendix 2-A

**Intended User**	**Intended Use**	**Format**	**Mental Health**	**Physical Health**	**General Health**	**Purpose**
FF = firefighter	FF = firefighter	1 = survey/questionnaire	0 = n/a or not stated	0 = n/a or not stated	0 = n/a or not stated	1 = prevention
FR = first responders	FR = first responders	2 = checklist	1 = PTSD	1 = cancer	1 = wellbeing/safety	2 = measuring exposure
FC = fire chiefs	FC = fire chiefs	3 = app	2 = critical incident stress	2 = musculoskeletal	2 = nutrition	3 = return to work
P = physicians of firefighters	P = physicians of firefighters	4 = information package	3 = suicide	3 = reproductive	3 = mindfulness	4 = maintaining health
	R = researcher	5 = forum	4 = substance abuse	4 = fitness	4 = other/possible but unconfirmed	5 = education/act as a resource
			5 = anxiety	5 = other/possible but unconfirmed		6 = networking
			6 = depression			
			7 = other/possible but unconfirmed			

Appendix 2-B: Table of Tools (links, date of access, date of release, access)

Developer	Tool Name	Link to Tool	Date Accessed	Date of Release/Update	Access
IAFF	Cancer registry	http://client.prod.iaff.org/#contentid=271	July 12, 2017	June 16, 2014	require login
IAFF	IAFF Frontlines App	https://itunes.apple.com/us/app/iaff-frontline/id884513271?mt=8	July 12, 2017	April 28, 2017	require login
launched by Canadian Association of Fire Chiefs, developed by Association of American Railroads	AskRail App	http://www.cn.ca/askrail - http://www.cafc.ca/news/260450/New-mobile-app-to-give-Canadian-first-responders-real-time-dangerous-goods-information-AskRail.htm	July 12, 2017	December 15, 2016	require login
IAFF, linked by Alberta Fire Chiefs Association	Terrorism Response Checklist	https://afca.ca/training/item/234-iafc-terrorism-response-checklist	July 12, 2017		open access
Centre for Suicide Prevention, linked by BC Professional FF	First Responders Trauma Intervention and Suicide Prevention	http://www.bcpffa.org/docs/CMHA%20-%20First%20Responders%20Toolkit.pdf	July 12, 2017	2015	open access
Newfoundland and Labrador Association of Fire Services	Member Forum	http://nlfireservicesforum.com/	July 12, 2017	July 5, 2017	require login
Ontario Association of Fire Chiefs	Job Performance Checklist for Firefighters	http://www.oafc.on.ca/job-performance-checklists http://www.oafc.on.ca/system/files/privateattachments/page/2861/Firefighter%20JPC.pdf	July 12, 2017	February 11, 2008	open access
Ontario Association of Fire Chiefs	Job Performance Checklist for Company Officers	http://www.oafc.on.ca/system/files/privateattachments/page/2861/Company%20Officer%20JPC.pdf	July 12, 2017	February 11, 2008	open access
Hamilton Professional FF Association	Xposure	http://news.engineering.utoronto.ca/multidisciplinary-capstone-project-app-helps-firefighters-track-hazard-exposure/	July 12, 2017	March 4, 2016	open access
Toronto Professional FF Association	Member Forum	https://www.torontofirefighters.org/login/?redirect_to=https://www.torontofirefighters.org/forums/	July 12, 2017	unknown	require login
Greater Sudbury Professional FF Association	Member Forum	http://www.iaff527.org/login/index.cfm?toUrl=%2Fforums%2Fone%2Findex%2Ecfm%3F&CFID=25381635&CFTOKEN=31745390	July 12, 2017	unknown	require login
Norfolk Professional FF Association	Member Forum	http://www.iafflocal68.org/login/index.cfm?toUrl=%2Fforums%2Fone%2Findex%2Ecfm%3F&CFID=25573293&CFTOKEN=99813140	July 12, 2017	unknown	require login

Developer	Tool Name	Link to Tool	Date Accessed	Date of Release/Update	Access
Ottawa Professional FF Association	PAR-Q and You	http://www.ottawafirefighters.org/items/par-q_FINAL.pdf	July 12, 2017	2002	open access
Ottawa Professional FF Association	Firefighters Medical Checklist	http://www.ottawafirefighters.org/items/Individualized_Health_Risk_Appraisal_V1%203.pdf	July 12, 2017	2007	open access
Oshawa Professional FF Association	Member Forum	http://www.iaff465.com/login/index.cfm?toUrl=%2Fforums%2Fone%2Findex%2Ecfm%3F&CFID=10476119&CFTOKEN=50287233	July 12, 2017	unknown	require login
Clarington Professional FF Association	Member Forum	http://www.iaff465.com/login/index.cfm?toUrl=%2Fforums%2Fone%2Findex%2Ecfm%3F&CFID=10476119&CFTOKEN=50287233	July 12, 2017	unknown	require login
Whitby Professional FF Association	Member Forum	http://www.iaff2036.org/login/index.cfm?toUrl=%2Findex%2Ecfm%3Fsection%3D24%26pagenum%3D129&CFID=59341218&CFTOKEN=46034732	July 12, 2017	unknown	require login
Waterloo Professional FF Association	Member Forum	http://www.waterloofirefighters.org/login/index.cfm?toUrl=%2Fforums%2Fone%2Findex%2Ecfm%3F&CFID=59488219&CFTOKEN=56176873	July 12, 2017	unknown	require login
United Firefighters of Winnipeg	CISM Peer Support Team Contact List	http://www.wfpsonline.com/documents/wellness/CISM_Peer_Support_Team_Contact_List.pdf	July 12, 2017	May 31, 2017	open access
Sault Ste. Marie Professional Firefighters Association	Exposure Report	http://www.saultfirefighters.ca/form.cfm?id=18	July 14, 2017	unknown	open access
