# Supplementary material for: Health Information on Firefighter Websites: Structured Analysis
Source: Interact J Med Res. 2018 Jul 16;7(2):e12. doi: 10.2196/ijmr.9369 (PMC6066636; doi:10.2196/ijmr.9369)
Supplement: Multimedia Appendix 3 [file ijmr_v7i2e12_app3.pdf]

Appendix 3-A: IAFF Mental Health Resources Table (description, intended audience, type, format, and focus of resource)

Resource Name	Description	Intended Audience	Type of Resource	Format	Focus
Peer Support Training	give FF the knowledge and skills to support their peers through mental health challenges	firefighters	1	1	4
Online Behavioral Health Awareness Course	covers topics including depression, substance abuse, trauma, and PTSD, and strategies for maintaining balance and emotional wellness	firefighters	1	1	4
IAFF Centre of Excellence for Behavioural Health Treatment and Recovery Webinar	professionals answer questions on the access, expectations, and treatment for FF who are affected by addiction or mental disorders such as PTSD, and would like to seek treatment at the in-patient residential facility	firefighters	2	3	4
Addressing PTSD Through a Peer Support Program	advantages of using peer support programs for PTSD	firefighters fire chiefs	2	3	1
Post Traumatic Stress Disorder in the Fire Service	causes and signs/symptoms of PTSD and best practices for addressing it within your local	firefighters	2	3	1
"Bringing PTSD Out of the Shadows" from 2015 Fire Fighter Quarterly Magazine	magazine article about addressing PTSD	firefighters	2	6	1
Suicide Postvention SOP Project	info sheet/guideline on steps to recover after a FF suicide	firefighters fire chiefs	2	1	5
Suicide Awareness Resources and Materials	links to suicide information and contacts to access more resources	firefighters	1	1	5
Responding to Members' Behavioral Health Needs: A Three Month Action Plan for IAFF Leaders	outline for leaders within fire departments to meet the behavioural health needs of its members by setting up support programs, professional contacts such as clinicians	fire chiefs	2	4	4
Firestrong Website link	comprehensive website with many resources and links to information pages, contact info for people who can provide support 24/7	firefighters	2	7	4
National Centre for PTSD link	website with many resources to learn about research findings re: PTSD	firefighters	2	7	1
National Suicide Prevention Lifeline link	phone number for support for people in a crisis	anyone at risk of suicide	2	9	5

Legend for Appendix 3-A

**Level**	**Type of Organization**	**Type of Resource**	**Format**	**Focus**
1 = national	1 = employer (IAFF/fire chiefs association/city)	1 = info on in-person course	1 = factsheet	1 = PTSD
2 = provincial	2 = employee (volunteer/professional FF association)	2 = immediate information and reference	2 = infographic/poster	2 = critical incident stress
3 = municipal			3 = video	3 = addiction/substance abuse
			4 = guide/manual	4 = general mental health
			5 = brochure/info packet	5 = suicide
			6 = article	6 = other
			7 = general website	
			8 = study/report	
			9 = other	

Appendix 3-B: IAFF Mental Health Resources Table (link, purpose, date accessed, and accessibility)

Resource Name	Link to Resource	Purpose of Resource	Date Accessed	Access
Peer Support Training	http://client.prod.iaff.org/#contentid=40484	info sheet on course info and eligibility for interested IAFF members	July 18, 2017	course only for IAFF members
Online Behavioral Health Awareness Course	http://client.prod.iaff.org/#page=behavioralhealth	online course through login	July 18, 2017	course only for IAFF members
IAFF Centre of Excellence for Behavioural Health Treatment and Recovery Webinar	https://www.youtube.com/watch?v=BC_od7bsNW4&feature=youtu.be	have common questions answered by professionals	July 18, 2017	open access
Addressing PTSD Through a Peer Support Program	https://www.youtube.com/watch?v=CTHBtB92uDA	encourage the implementation of peer support groups	July 18, 2017	open access
Post Traumatic Stress Disorder in the Fire Service	https://www.youtube.com/watch?v=WkzztWPuto8	provide info	July 18, 2017	open access
"Bringing PTSD Out of the Shadows" from 2015 Fire Fighter Quarterly Magazine	http://www.iaff.org/mag/2015/01/html5/	reduce the stigma of PTSD	July 18, 2017	open access
Suicide Postvention SOP Project	http://client.prod.iaff.org/#contentid=1749	resources for after suicide of a FF	July 18, 2017	open access
Suicide Awareness Resources and Materials	http://client.prod.iaff.org/#contentid=1748	raise awareness and prevent suicide	July 18, 2017	open access (some need login to access)

Resource Name	Link to Resource	Purpose of Resource	Date Accessed	Access
Responding to Members' Behavioral Health Needs: A Three Month Action Plan for IAFF Leaders	http://services.prod.iaff.org/ContentFile/Get/30684	provide resource for fire departments looking to create an action plan	July 18, 2017	open access
Firestrong Website link	https://www.firestrong.org/	provide resources	July 18, 2017	open access
National Centre for PTSD link	https://www.ptsd.va.gov/	provide resources	July 18, 2017	open access
National Suicide Prevention Lifeline link	https://suicidepreventionlifeline.org/	provide immediate support over the phone	July 18, 2017	open access
