# Supplementary material for: Health Information on Firefighter Websites: Structured Analysis
Source: Interact J Med Res. 2018 Jul 16;7(2):e12. doi: 10.2196/ijmr.9369 (PMC6066636; doi:10.2196/ijmr.9369)
Supplement: Multimedia Appendix 4 [file ijmr_v7i2e12_app4.pdf]

Appendix 4-A: IAFF Physical Health Resources Table (description, intended audience, type, format, and focus of resource)

Resource Name	Description	Intended Audience	Type of Resource	Format	Focus
Peer Fitness Trainer Certification Program	FF trained with skills to set up and lead fitness program for other FF in their own department	fire chiefs	1	1	5
Burn Injury Assistance	providing FF and their immediate family with financial assistance after a burn injury	firefighters	2	1	2
Cancer Awareness and Prevention Resource	Understand your cancer risk, know the exposures to carcinogens on the job and reduce your risk of occupational cancer	firefighters	1	1	1
Firefighter Cancer Support Network link	link to general website created to support FF with cancer	firefighters	2	7	1
Cancer Awareness	providing info on cancers common in FF	firefighters	2	3	1
Fit to Survive: the firefighter's guide to health and nutrition	provide nutrition and health advice	firefighters	2	7	5
Line of Duty Death Resources	list of linked resources, including data bases, manuals, info sheets on policies	fire chiefs	2	1	7
Presumptive Health Initiative	summaries of each province/territory/state's presumptive health legislations	firefighters	2	1	7
World Trade Centre Health link	division of IAFF website for first responders who were involved in the 9/11 attacks; resource for compensation funds and support programs	firefighters	2	7	7
Infectious Diseases Resource	division of IAFF website with comprehensive access to resources regarding infectious diseases (control practices, principles, and detailed info on diseases)	firefighters	2	7	6
National Institute for Occupational Safety and Health: Health Hazard Evaluation Program	info sheet on service for identifying hazards in workplace	fire chiefs	1	1	6
Vaccine Preventable Diseases	list of vaccine preventable diseases and recommended vaccine schedule	firefighters	2	1	6

Resource Name	Description	Intended Audience	Type of Resource	Format	Focus
Campaign for a Smoke Free Union	division of IAFF website offering resources and programs to aid FF in quitting smoking	firefighters	2	7	6
A Labor Perspective on Workplace Reproductive Hazards	report of a study explaining reproductive hazards across all occupations and ways to prevent harm	firefighters	2	8	6
Reproductive Hazards Policy	report explaining each fire department's duty to provide resources in the form of a designated physician responsible for guiding FF on their reproductive health, assisting pregnant FF	female firefighters	2	8	6
Reproductive Hazards in the Workplace: A Case Study on Women Firefighters	report on study looking at the potential faced by pregnant FF and the ideal policies to be put in place	female firefighters	2	8	6
Reproductive Hazards of Firefighting: Non-chemical Hazards	report on study looking at the effects of non-chemical hazards such as noise, heat, and physical exertion on reproductive health	firefighters	2	8	6
The Firefighting Environment Risk for Breast Cancer, Gynecologic Malignancies and Lymphoma	report summarizes the findings of the chemicals or substances found in the fire fighting environment that may be associated with cancers that affect women.	firefighters	2	8	6

Legend for Appendix 4-A

Level	Type of Organization	Type of Resource	Format	Focus
1 = national	1 = employer (IAFF/fire chiefs association/city)	1 = info on in-person course	1 = factsheet	1 = cancer
2 = provincial	2 = employee (volunteer/professional FF association)	2 = immediate information and reference	2 = infographic/poster	2 = burns
3 = municipal			3 = video	3 = musculoskeletal
			4 = guide/manual	4 = reproductive
			5 = brochure/info packet	5 = fitness
			6 = article	6 = health hazards
			7 = general website	7 = other

			8 = report	

Appendix 4-B: IAFF Physical Health Resources Table (link, purpose of resource, date accessed, accessibility)

Resource Name	Link to Resource	Purpose of Resource	Date Accessed	Access
Peer Fitness Trainer Certification Program	http://www.iaff.org/HS/PFT/PeerIndex.htm	allow leaders within fire departments to bring skills learned in course back to their own crew	July 18, 2017	only IAFF members can attend course
Burn Injury Assistance	http://client.prod.iaff.org/#contentid=1741	info on eligibility and application	July 18, 2017	only IAFF members are eligible
Cancer Awareness and Prevention Resource	http://client.prod.iaff.org/#contentid=40435	info on course	July 18, 2017	only IAFF members can attend course
Firefighter Cancer Support Network link	https://firefightercancersupport.org/	provide resources and support	July 18, 2017	open access
Cancer Awareness	https://www.youtube.com/watch?list=PLid8i4Cga6mDAFk43wZ0iwxPJnL0DUfdY&v=sYzZygH4m8M	raise awareness	July 18, 2017	open access
Fit to Survive: the firefighter's guide to health and nutrition	http://www.iaff.org/hs/FTS/ftsdefault.asp	provide resource, meal plan, and advice	July 18, 2017	open access
Line of Duty Death Resources	http://client.prod.iaff.org/#contentid=369	provide resources and support	July 18, 2017	open access
Presumptive Health Initiative	http://phi.iaff.org/Map.aspx	act as a resource	July 18, 2017	open access
World Trade Centre Health link	http://wtc.iaff.org/	act as a resource	July 18, 2017	open access
Infectious Diseases Resource	http://infdisease.iaff.org/	act as a resource	July 18, 2017	open access
National Institute for Occupational Safety and Health: Health Hazard Evaluation Program	http://www.iaff.org/hs/NIOSH_HHE.htm	identify hazards	July 18, 2017	service only for IAFF locals
Vaccine Preventable Diseases	http://www.iaff.org/hs/VaccinePreventableDiseases.htm	provide info on recommended vaccines	July 18, 2017	open access
Campaign for a Smoke Free Union	http://www.iaff.org/smokefree/	resources to quit smoking	July 18, 2017	open access
A Labour Perspective on Workplace Reproductive Hazards	http://services.prod.iaff.org/ContentFile/Get/20217	raise awareness of workplace hazards	July 18, 2017	open access

Resource Name	Link to Resource	Purpose of Resource	Date Accessed	Access
Reproductive Hazards Policy	http://services.prod.iaff.org/ContentFile/Get/20218	raise awareness of workplace hazards	July 18, 2017	open access
Reproductive Hazards in the Workplace: A Case Study on Women Firefighters	http://services.prod.iaff.org/ContentFile/Get/20220	raise awareness of workplace hazards	July 18, 2017	open access
Reproductive Hazards of Firefighting: Non-chemical Hazards	http://services.prod.iaff.org/ContentFile/Get/20221	raise awareness of workplace hazards	July 18, 2017	open access
The Firefighting Environment Risk for Breast Cancer, Gynecologic Malignancies and Lymphoma	http://services.prod.iaff.org/ContentFile/Get/20219	raise awareness of workplace hazards	July 18, 2017	open access
