# Supplementary material for: Health Information on Firefighter Websites: Structured Analysis
Source: Interact J Med Res. 2018 Jul 16;7(2):e12. doi: 10.2196/ijmr.9369 (PMC6066636; doi:10.2196/ijmr.9369)
Supplement: Multimedia Appendix 5 [file ijmr_v7i2e12_app5.pdf]

Appendix 5-A: BC Mental Health Resources Table (description, intended audience, type of resource, format and focus)

Resource Name	Description	Intended Audience	Type of Resource	Format	Focus
Best Practices for Mental Health in the Fire Service	document from the BCPFFA Mental Health Task Force outlining 6 areas that need improvement, and instructions on the implementation of programs to address the gaps	fire chiefs	2	5	4
Caring for Yourself and Your Colleaugues in the Fire Service	document for firefighters raising awareness of suicide, common myths, the impact of suicide, and when/how to get help	firefighters	2	5	5
Myths about Suicide (WHO link)	series of myths about suicide followed by an explanation	firefighters	2	2	5
Preventing Suicide	info on how to help someone thinking about suicide, how to reduce the risk, and avenues for help	firefighters	2	5	5
Coping with Suicidal Thoughts	info on creating a safety plan, potential causes for thoughts of suicide, and help centres	anyone with suicidal thoughts	2	5	5
Suicide Awareness	handout to FF to provide info about suicide, warning signs, and when to get help	firefighters	2	5	5
Seeking Help	poster with 24 hour crisis line contact info; making it clear that reaching out for help is not a sign of weakness	firefighters	2	2	5
Preventing Suicide (WHO link)	comprehensive resource for understanding and preventing suicide	firefighters, fire chiefs	2	4	5
Joint Mental Health and Wellness Committee	BCPFFA's recommendations to each municipal fire department to form a mental health and wellness committee	fire chiefs	2	1	5
British Columbia Association of Fire Chaplains link	brochure highlighting the role of fire chaplains in the fire service	fire chiefs	2	5	4
First Responders Trauma Intervention and Suicide Prevention Resource Toolkit	comprehensive resource for understanding and preventing suicide	firefighters	2	4	5
Critical Incident Stress Management Operational Guideline Template	template for fire departments looking to establish their own CISM teams	fire chiefs	2	9	2

Resource Name	Description	Intended Audience	Type of Resource	Format	Focus
CISM Team Application	form for FF interested in joining the CISM team in their department to fill out to apply for the position	firefighters	2	9	2
CISM Defusing Checklist	checklist for procedure of a CISM debrief session	firefighters	2	9	2
CISM Handout - Member	given to FF after a potentially traumatic call with info on signs and symptoms how to get help	firefighters	2	5	2
CISM Handout - Family Member	given to family members of FF on how best to support them after a critical incident	firefighters' family	2	5	2
Spousal Critical Incident Stress Management (CISM) Manual	comprehensive resource for spouses of FF	firefighter's spouse	2	4	2

Legend for Appendix 5-A:

Level	Type of Organization	Type of Resource	Format	Focus
1 = national	1 = employer (IAFF/fire chiefs association/city)	1 = info on in-person course	1 = factsheet	1 = PTSD
2 = provincial	2 = employee (volunteer/professional FF association)	2 = immediate information and reference	2 = infographic/poster	2 = critical incident stress
3 = municipal			3 = video	3 = addiction/substance abuse
			4 = guide/manual	4 = general mental health
			5 = brochure/info packet	5 = suicide
			6 = article	6 = other
			7 = general website	

			8 = study/report	
			9 = other	

Appendix 5-B: BC Mental Health Resources Table (link, purpose, date accessed, and accessibility)

Resource Name	Link to Resource	Purpose of Resource	Date Accessed	Access
Best Practices for Mental Health in the Fire Service	http://www.bcpffa.org/docs/Final%20-%20BCPFFA%20Mental%20Health%20Task%20Force%20-%20Best%20Practices%20Recommendations.pdf	outlines the issues discovered through a province wide survey and details ways to improve mental health in FF	Jul 19, 2017	open access
Caring for Yourself and Your Colleaugues in the Fire Service	http://www.bcpffa.org/docs/Fire%20Personnel%20Handout%20-Suicide%20%28EAP%29.pdf	resource for FF in hopes of preventing suicide	Jul 19, 2017	open access
Myths about Suicide (WHO link)	http://www.bcpffa.org/docs/Myths%20about%20suicide.pdf	details the misconceptions about suicide attempting to allow people thinking about suicide to reach out for help	Jul 19, 2017	open access
Preventing Suicide	http://www.bcpffa.org/docs/Preventing-Suicide-NTNL-brochure-2014-web.pdf	prevention of suicide	Jul 19, 2017	open access
Coping with Suicidal Thoughts	http://www.bcpffa.org/docs/Coping_with_suicidal_thoughts_brochure_-_BC.PDF	resource for better understanding suicidal thoughts	Jul 19, 2017	open access
Suicide Awareness	http://www.bcpffa.org/docs/Suicide%20Awareness%20Handout.pdf	resource for firefighters to raise awareness of suicide	Jul 19, 2017	open access
Seeking Help	http://www.bcpffa.org/docs/BCPFFA%20Seeking%20Help%20Poster.pdf	giving FF people to contact for help	Jul 19, 2017	open access
Preventing Suicide (WHO link)	http://www.bcpffa.org/docs/PREVENTING%20SUICIDE%20-%20WHO%20.pdf	resource	Jul 19, 2017	open access
Joint Mental Health and Wellness Committee	http://www.bcpffa.org/docs/BCPFFA%20Mental%20Health%20Task%20Force%20-%20Mental%20Health%20and%20Wellness%20Committee%20Template.pdf	encouraging mental health to be addressed in municipal fire departments	Jul 19, 2017	open access

**Resource Name**	**Link to Resource**	**Purpose of Resource**	**Date Accessed**	**Access**
British Columbia Association of Fire Chaplains link	http://www.bcpffa.org/docs/BCAFC%20Brochure%20Chaplaincy.pdf	providing info on what a fire chaplain can provide to a fire department	Jul 19, 2017	
First Responders Trauma Intervention and Suicide Prevention Resource Toolkit	http://www.bcpffa.org/docs/CMHA%20-%20First%20Responders%20Toolkit.pdf	education	Jul 19, 2017	open access
Critical Incident Stress Management Operational Guideline Template	http://www.bcpffa.org/docs/BCPFFA%20Mental%20Health%20Task%20Force%20-%20CISM%20OG%20TEMPLATE.docx	make it easier for departments to create their own CISM team	Jul 19, 2017	open access
CISM Team Application	http://www.bcpffa.org/docs/CISM%20Team%20Application%20and%20Waiver%20Form.docx	form to submit application	Jul 19, 2017	open access
CISM Defusing Checklist	http://www.bcpffa.org/docs/Basic%20CISM%20Checksheet%20and%20Record.docx	make sure all steps are followed in the correct order	Jul 19, 2017	open access
CISM Handout - Member	http://www.bcpffa.org/docs/CISM%20Signs%20and%20Symptoms%20-%20Member%20Handout.docx	provide info	Jul 19, 2017	open access
CISM Handout - Family Member	http://www.bcpffa.org/docs/CISM%20Signs%20and%20Symptoms%20-%20Family%20Handout.docx	provide info	Jul 19, 2017	open access
Spousal Critical Incident Stress Management (CISM) Manual	http://www.bcpffa.org/docs/Spousal%20CISM%20Booklet%20%26%20Information%20Sheets.doc	act as a resource	Jul 19, 2017	open access
